# Photoprocesses in Bis-Diethylamino Derivatives of 1,4- and 1,3-Distyrylbenzene

**DOI:** 10.3390/molecules29174139

**Published:** 2024-08-31

**Authors:** Levon S. Atabekyan, Vitaly G. Avakyan, Marina V. Fomina, Vyacheslav N. Nuriev, Alexey V. Medved’ko, Sergey Z. Vatsadze, Sergey P. Gromov, Alexander K. Chibisov

**Affiliations:** 1 NRC “Kurchatov Institute”, Kurchatov Complex of Crystallography and Photonics, Photochemistry Center, Novatorov Str. 7A-1, Moscow 119421, Russialexeym@gmail.com (A.V.M.); spgromov@mail.ru (S.P.G.);; 2 Department of Chemistry, M. V. Lomonosov Moscow State University, Build. 3, 1 Leninskie Gory, Moscow 119991, Russia; 3 N.D. Zelinsky Institute of Organic Chemistry, Russian Academy of Sciences, 47 Leninskii Prospekt, Moscow 119991, Russia

**Keywords:** distyrylbenzene, fluorescence, laser photolysis, triplet–triplet dismutation, radical species

## Abstract

The photoprocesses of diethylamino derivatives of 1,4- and 1,3-distyrylbenzenes in MeCN were studied using absorption, luminescence, ^1^H NMR, and laser kinetic spectroscopy. Compounds **1** and **2** undergo intersystem crossing to the triplet state and exhibit delayed fluorescence. It was concluded that dye radical anions and radical cations are formed upon dismutation of the triplet state in the presence of an electron donor or acceptor. Quantum mechanical calculations for the structures of diethylamino distyrylbenzenes in the ground and excited states were carried out, making it possible to establish the structures of isomers and the most stable conformers for both compounds.

## 1. Introduction

Distyrylbenzenes are photoactive compounds applicable for the design of organic light-emitting diodes, solar cells, nonlinear-optical materials, and chemical sensors [1,2,3,4,5,6]. Owing to the presence of two C=C double bonds, distyrylbenzene molecules are characterized by light sensitivity, which provides additional possibilities for their phototransformations. Distyrylbenzenes are addressed in quite a few publications [7,8,9,10,11,12,13,14,15,16], but no data on the study of photoprocesses in distyrylbenzenes and their derivatives are available. Previously, we investigated the photonics of 1,4- and 1,3-diazadistyrylbenzene derivatives containing quaternary nitrogen atoms as strong electron-withdrawing moieties [17] and showed that the photoprocesses characteristics of the 1,4- and 1,3-diazadistyrylbenzene isomers are considerably different.

**Figure 1:**
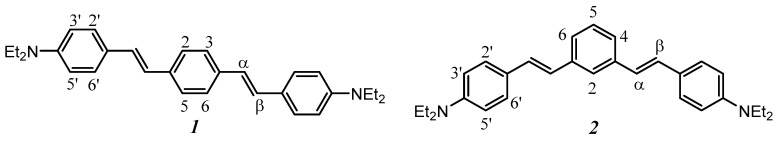
Structures of diethylamino 1,4- and 1,3-distyrylbenzenes (**1**, **2**).

The phototransformations of 1,4-diazadistyrylbenzene derivatives include *trans*–*cis* isomerization and the intersystem crossing to the triplet state. In this case, the formation of dihydrophenanthrene (DHP) is energetically unfavorable. For 1,3-diazadistyrylbenzene, the *trans–cis* isomerization is followed by electrocyclization of the *cis*–form to DHP and the subsequent fast oxidation with oxygen to give phenanthrene, whereas the intersystem crossing to the triplet state is not effective. This study is devoted to 1,4- and 1,3-distyrylbenzene derivatives containing strong electron-donating diethylamino groups. The structures with atom numbering differing from the IUPAC rules of the studied distyrylbenzenes are shown in Figure 1.

The purpose of this study is to compare the photonics of compounds in MeCN using absorption, fluorescence, and laser kinetic spectroscopy, and to establish the presence of the triplet state and intermediate products of photoreactions. The experiments are accompanied by quantum chemical calculations in order to determine the chromophore structures in the ground and excited states and to interpret the experimental absorption and fluorescence spectra. ^1^H NMR spectroscopy was used to study the *trans–cis* photoisomerization of diethylamino 1,4- and 1,3-distyrylbenzenes.

## 2. Results and Discussion

### 2.1. Electronic Absorption and Fluorescence Spectra

**Figure 2:**
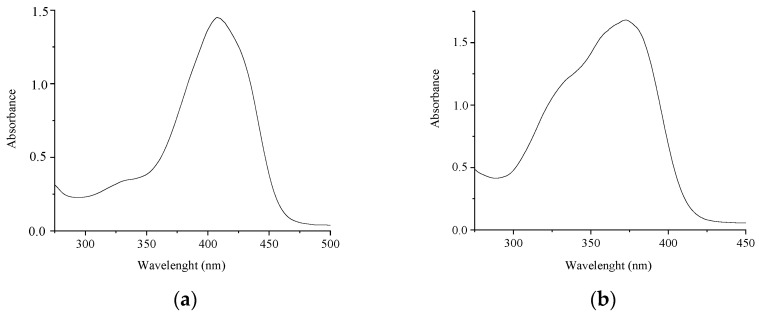
Absorption spectra of compounds **1** (**a**) and **2** (**b**) in MeCN. The concentrations of the compounds are 4 × 10^−5^ M (**a**) and 2.5 × 10^−5^ M (**b**).

Figure 2 shows the absorption spectra of compounds **1** and **2**. The spectra of **1** and **2** differ in both the shape and positions of the absorption maxima. The absorption band of **2** is considerably blue-shifted (by 40 nm) compared to that of **1**. The experimental and calculated absorption maxima are given in Table 1.

**Figure 3:**
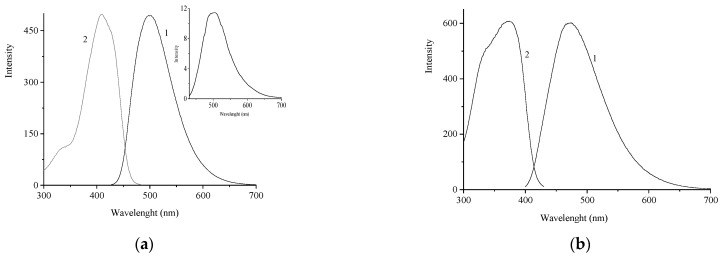
Fluorescence (1) and fluorescence excitation (2) spectra of compound **1** (**a**) and compound **2** (**b**) in MeCN. The inset of (**a**) shows the delayed fluorescence spectra of **1**. The concentration of compounds is 5 × 10^−6^ M.

Figure 3 shows the fluorescence spectra of **1** and **2**, while Table 2 summarizes the absorption maxima, Stokes shifts, and fluorescence quantum yields.

Fluorescence is observed for both **1** and **2**. The fluorescence excitation spectra coincide with the absorption spectra of these compounds. For compound **2**, there is a large Stokes shift of the fluorescence band (100 nm) and a considerable (almost 2.5-fold) decrease in the fluorescence quantum yield compared to **1**; this may be attributable to a lower degree of conjugation in **2**. Both compounds show P-type delayed fluorescence, indicating the intersystem crossing to the triplet state. The presence of P-type fluorescence is evidenced by the lack of temperature dependence of the delayed fluorescence intensity.

### 2.2. Kinetic Spectroscopy

**Figure 4:**
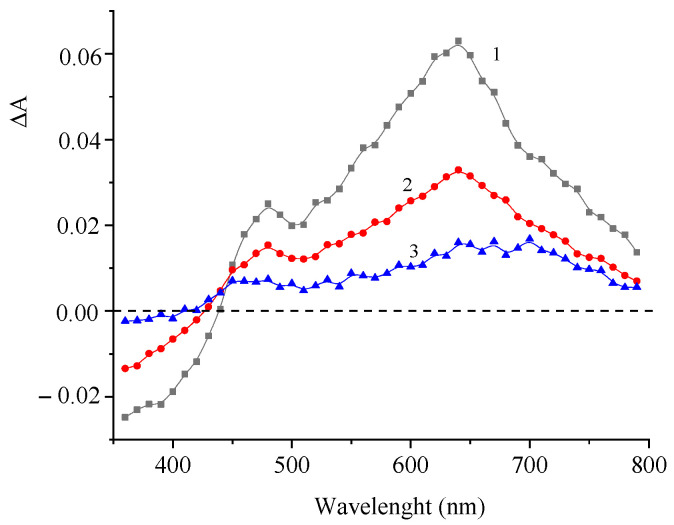
Difference photoinduced absorption spectra of a deoxygenated solution of **1** measured 2 (1), 15 (2), and 150 (3) µs after the laser pulse. The concentration of **1** is 5 × 10^−5^ M.

Pulsed laser excitation of compound **1** results in a change in the absorption spectrum, as shown in Figure 4.

**Figure 5:**
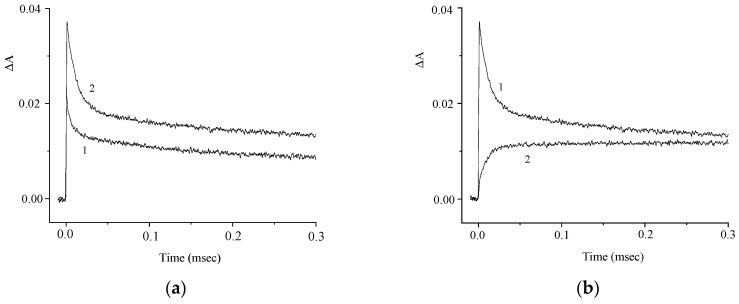
Kinetic curves of the absorbance change at λ = 630 nm in (**a**) air-saturated (1) and deoxygenated (2) solutions of **1**; (**b**) deoxygenated solution of **1** at λ = 630 nm (curve 1) and 440 nm (curve 2). The concentration of **1** is 5 × 10^−5^ M.

The difference spectrum exhibits a decrease in the absorbance in the main absorption band of the dye (350–440 nm) and an increase in the absorbance in the 450–800 nm range with a maximum at 640 nm as a result of the formation of short-lived photoreaction products. Figure 5 presents the corresponding kinetic curves measured at the photoinduced absorption maxima.

**Figure 6:**
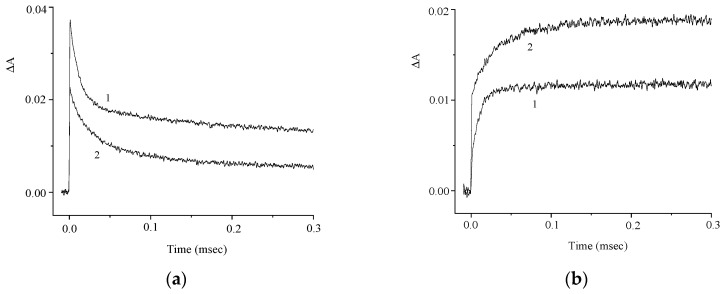
Kinetic curves of the absorbance change in a deoxygenated solution of **1** in the absence (curve 1) and in the presence (curve 2) of *para*-nitroacetophenone (PNAP); λ = 630 nm (**a**) and 440 nm (**b**). The concentration of **1** is 5 × 10^−5^ M, and that of PNAP is 1.2 × 10^−3^ M.

**Figure 7:**
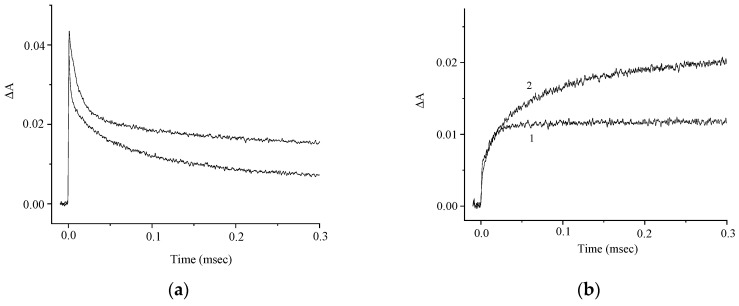
Kinetic curves of the absorbance change in a deoxygenated solution of **1** in the absence (curve 1) and in the presence (curve 2) of ascorbic acid (AA); λ = 630 nm (**a**) and 440 nm (**b**). The concentration of **1** is 5 × 10^−5^ M, and that of AA is 1 × 10^−3^ M.

The kinetic curves in Figure 5a demonstrate the effect of oxygen on the photoinduced absorption and attest to the presence of the intersystem crossing to the triplet state. The kinetics of the absorbance at λ = 630 nm is biexponential and is characterized by rate constants k_1_ = 9 × 10^4^ and k_2_ = 6 × 10^3^ s^−1^. The absorbances at λ = 630 nm and 440 nm vary in a similar way (Figure 5b), which attests to the formation of a photoproduct from the triplet excited state. In order to elucidate the nature of this photoproduct, we studied the effects of electron donors and acceptors on the kinetics of the phototransformations of **1**. Figure 6 and Figure 7 show the kinetic curves for absorption measured in the absence of, and in the presence of an electron acceptor [*para*-nitroacetophenone (PNAP)] and a donor [ascorbic acid (AA)], respectively. The effects of PNAP and AA on the kinetics of the photoinduced absorption lead to the conclusion that electron transfer occurs, producing the radical cation and the radical anion of compound **1**, respectively.

In the presence of PNAP and AA, the time for the relatively fast changes in the induced absorption decreases, while the time for the relatively slow decrease in the induced absorption at λ = 630 nm increases, along with a similar increase in the absorption at λ = 440 nm.

The observed results may be interpreted by assuming that the electron transfer, which results in the formation of radical species, occurs either due to the dismutation of molecules in the triplet state or due to the reaction of the triplet molecules with the dye molecules in the ground state [18,19,20]:

T + T → R^•−^ + R^•+^ (1)

T + S → R^•−^ + R^•+^ (2)

The fast decrease in the absorbance at 630 nm can be attributed to the R^•−^ radical, whose lifetime is reduced in the presence of oxygen, while the relatively slow stage can be associated with the lifetime of the R^•+^ radical. The increase in the duration of photoproduct accumulation at 440 nm, accompanied by a parallel decrease in the absorption at 630 nm, is attributable to the formation of radical cations and radical anions in the presence of an electron donor (D) and acceptor (A) when they react with the dye molecule in the triplet state [20,21].

A + T → R^•+^ + A^−^ (3)

D + T → R^•−^ + D^+^ (4)

Thus, in the presence of high concentrations of an electron donor or acceptor, reaction (1) or (2) is suppressed, while reaction (3) or (4) takes place. The observed photochemical transformations attest to the similarity between the absorption spectra of the dye radical cations and radical anions.

**Figure 8:**
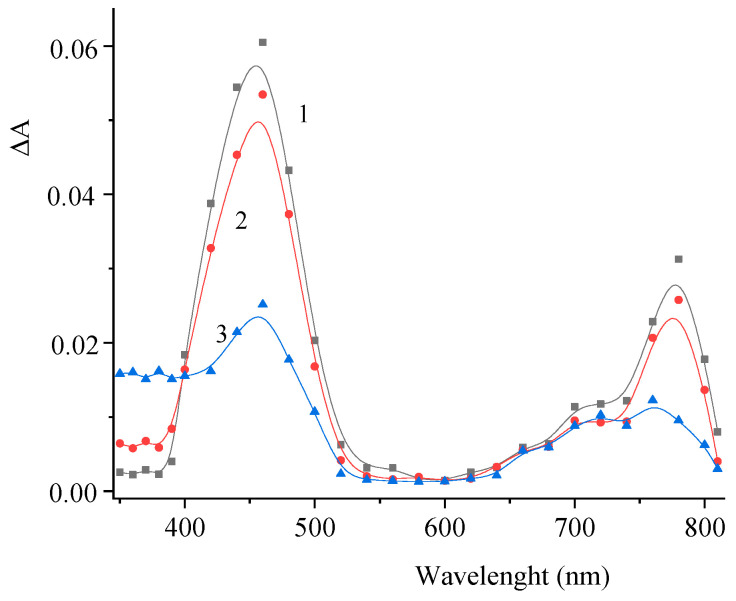
Difference photoinduced absorption spectra of a deoxygenated solution of **2** measured 3 (1), 10 (2), and 200 (3) µs after the laser pulse. The concentration of **2** is 3 × 10^−5^ M.

The pulsed laser excitation of compound **2** induces a change in the absorption spectrum depicted in Figure 8.

The difference spectrum shows an increased absorbance over the whole spectral range as a result of the formation of short-lived photoreaction products. The absence of a decrease in the absorbance at the major absorption band of the dye can be explained by the formation of a photoproduct with an absorption spectrum similar to that of **2**, which may correspond to the *cis*-isomer of the dye.

**Figure 9:**
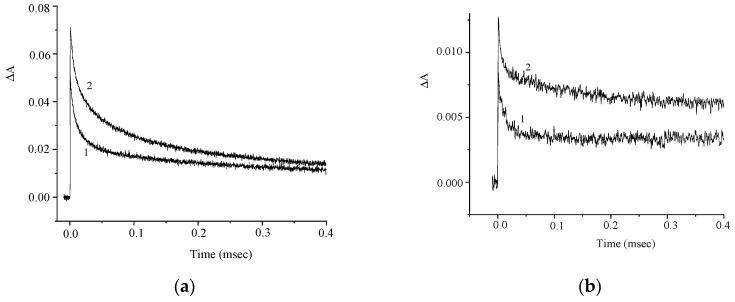
Kinetic curves of the absorbance change in air-saturated (1) and deoxygenated (2) solutions of **2** at λ = 460 nm (**a**) and 700 nm (**b**). The concentration of **2** is 2.5 × 10^−5^ M.

Figure 9 shows the corresponding kinetic curves measured at the photoinduced absorption maxima. The effect of air oxygen on the photoinduced absorption kinetics supports the conclusion that intersystem crossing to the triplet state of the dye occurs.

**Figure 10:**
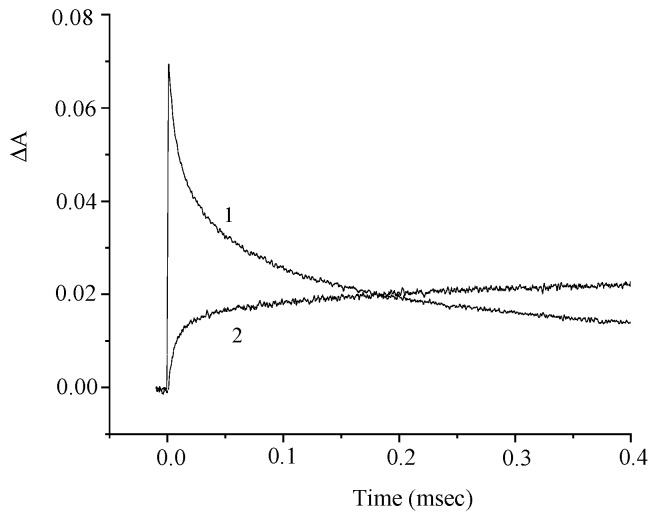
Kinetic curves of the absorbance change in a deoxygenated solution of **2** at λ = 460 nm (1) and 360 nm (2). The concentration of **2** is 2.5 × 10^−5^ M.

The kinetic curves presented in Figure 10 indicate a similar variation of the induced absorption at λ = 460 and 360 nm.

**Figure 11:**
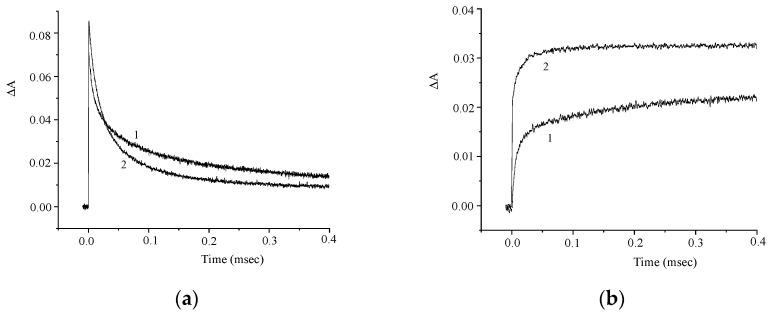
Kinetic curves of the absorbance change in a deoxygenated solution of **2** in the absence (curve 1) and in the presence (curve 2) of *para*-nitroacetophenone (PNAP). λ = 460 nm (**a**) and 360 nm (**b**). Concentrations: **2**, 2.5 × 10^−5^ M; PNAP, 6 × 10^−4^ M.

**Figure 12:**
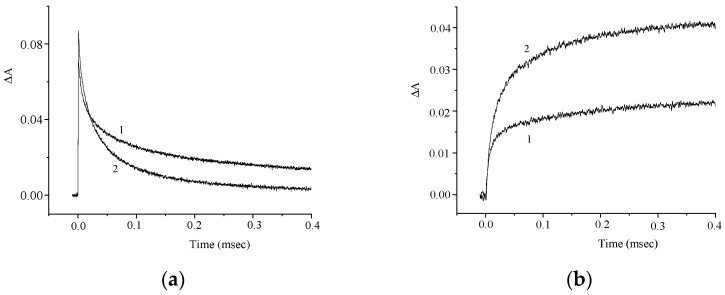
Kinetic curves of the absorbance change in a deoxygenated solution **2** in the absence (curve 1) and in the presence (curve 2) of ascorbic acid (AA). λ = 460 nm (**a**) and 360 nm (**b**). Concentrations: **2**, 2.5 × 10^−5^ M; AA, 5 × 10^−4^ M.

Similar to compound **1**, we can assume that T–T dismutation reaction takes place to give radical cations and radical anions of the dye (Reaction (1)). The effects of the electron donor (AA) and acceptor (PNAP) on the photoreaction kinetics are depicted in Figure 11 and Figure 12.

Similar to compound **1**, the experimental results can be interpreted by assuming that an electron acceptor or electron donor, when present in high concentration, reacts with the triplet molecule of dye **2** (Reactions (2) and (3)), thereby preventing the T–T dismutation (Reaction (1)).

### 2.3. ^1^H NMR Spectra

**Figure 13:**
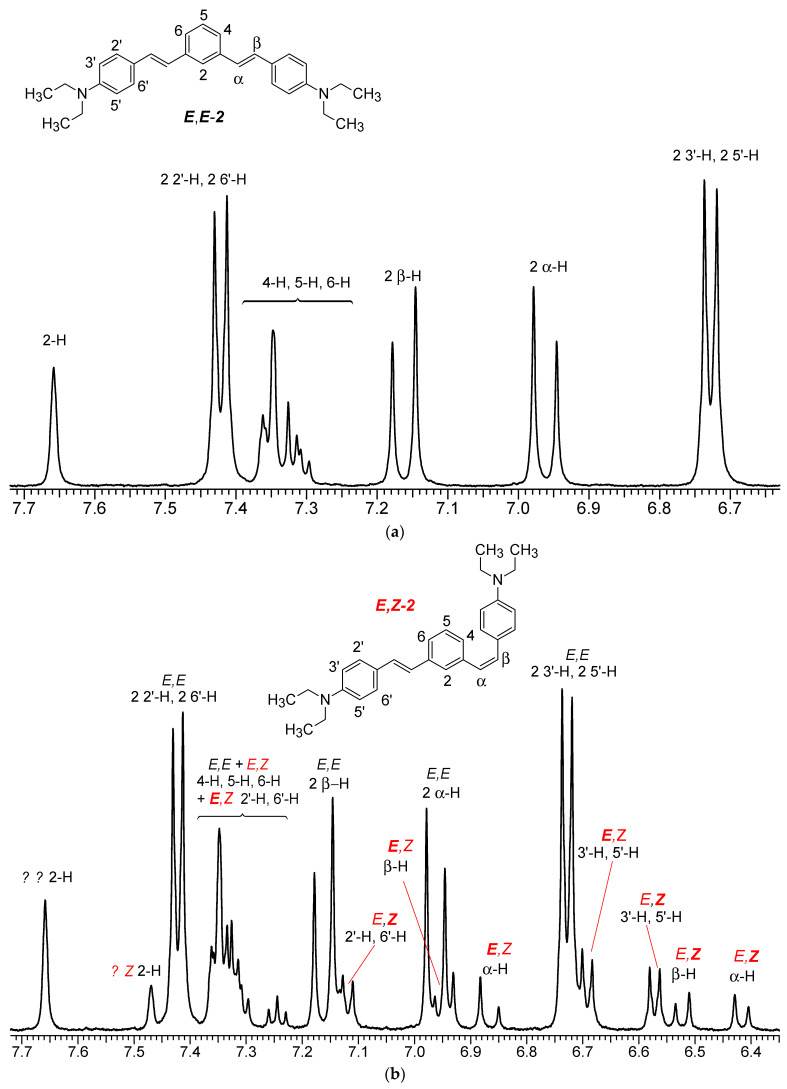
Aromatic proton region in the spectrum of 1,3-distyrylbenzene **2** before (**a**) and after (**b**) irradiation; 6.53–7.7 ppm range, MeCN-*d*_3_, 25 °C.

**Figure 14:**
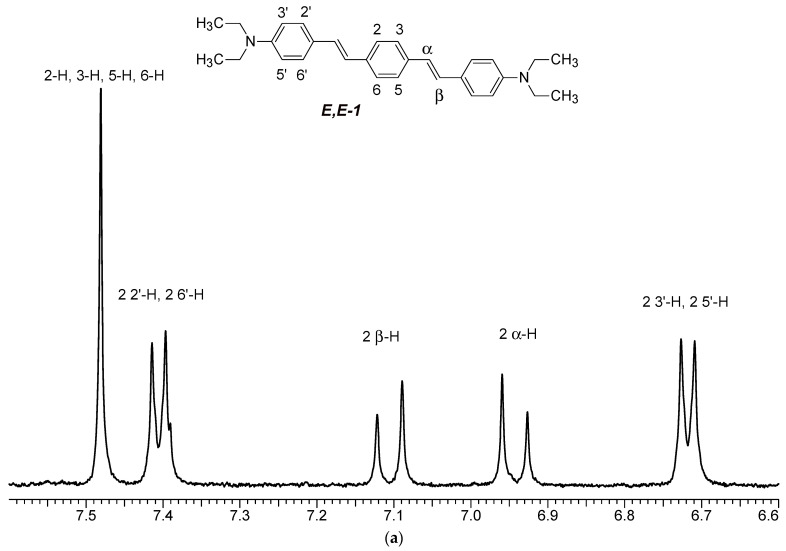

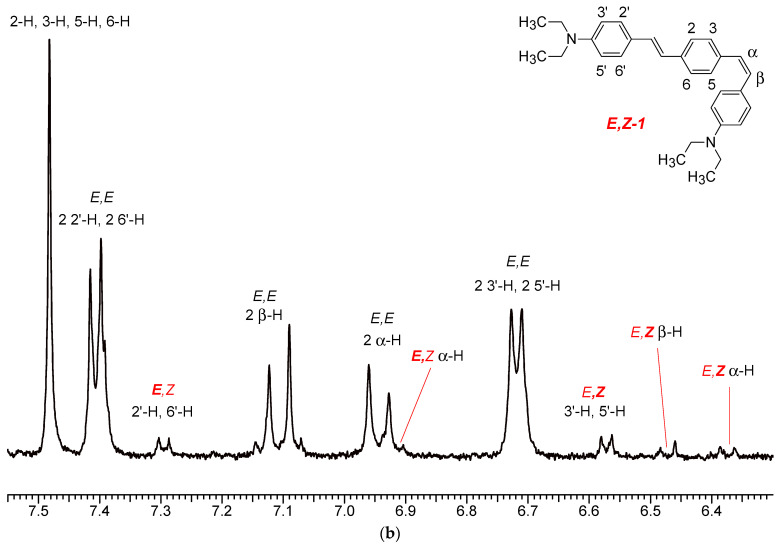
Aromatic proton region of the spectrum of 1,4-distyrylbenzene **1** before (**a**) and after (**b**) irradiation; 6.35–7.55 ppm range, MeCN-*d*_3_, 25 °C.

The ^1^H NMR spectra of distyrylbenzenes **2** and **1** (Figure 13a and Figure 14a) show one set of signals for each type of olefinic protons at 7.09–7.18 ppm for the β-H protons and at 6.93–6.98 ppm for the α-H protons, with spin–spin coupling constants of 16.3–16.4 Hz. These signals correspond to the (*E*,*E*)-isomers (proton numbering differing from the IUPAC rules is shown in Figure 13 and Figure 14). Data from the ^1^H NMR spectra indicate that distyrylbenzenes **1** and **2** were formed as (*E*,*E*)-isomers. After irradiation, the ^1^H NMR spectrum of 1,3-distyrylbenzene **2** (Figure 13b) exhibits three sets of signals for aliphatic, olefinic α-H and β-H protons, along with most aromatic protons, which indicates the formation of a mixture of (*E*,*Z*)- and (*E*,*E*)-isomers. In general, the proton chemical shifts of the *cis*-isomer are lower than those of the *trans*-isomer. The aliphatic part of the spectrum provides little information, as proton signals for the (*E*,*Z*)-isomer appear as multiplets and are superimposed on the signals of the (*E*,*E*)-isomer. The *cis*- and *trans*-isomers of unsaturated compounds can be unambiguously identified by their spin–spin coupling constants, which are much smaller for *cis*-interactions than for the *trans*-isomers. The α-H and β-H signals for the (*E*,*Z*)-isomer are observed more upfield. Indeed, α-H protons give rise to three doublets: 6.42 ppm (*J* = 12.2 Hz), 6.87 ppm (*J* = 16.3 Hz), both for the (*E*,*Z*)-isomer and an intense doublet at 6.97 ppm (*J* = 16.3 Hz) for the (*E*,*E*)-isomer. A similar pattern is observed for β-H protons, which give rise to three doublets: 6.53 ppm (*J* = 12.2 Hz), 6.95 ppm (*J* = 16.3 Hz), both for the (*E*,*Z*)-isomer, and an intense doublet at 7.17 ppm (*J* = 16.3 Hz) for the (*E*,*E*)-isomer. The 3′-H and 5′-H aromatic protons also appear as three doublets: 6.57 and 6.71 ppm for the (*E*,*Z*)-isomer and an intense doublet at 6.73 ppm for the (*E*,*E*)-isomer. The 2′-H and 6′-H protons give rise to a doublet at 7.12 ppm for the (*E*,*Z*)-isomer. The second signal corresponding to the (*E*,*Z*)-isomer cannot be unambiguously identified, as it is located more downfield as part of a complex multiplet formed by the signals of the 4-H, 5-H, and 6-H aromatic protons of the central benzene ring for both isomers. The third intense doublet corresponding to the 2′-H and 6′-H protons of the (*E*,*E*)-isomer occurs at 7.42 ppm. The 2-H aromatic protons appear as two singlets at 7.47 and 7.66 ppm for (*E*,*Z*)- and (*E*,*E*)-isomers, respectively. Analysis of the integral intensities of proton signals corresponding to the (*E*,*E*)- and (*E*,*Z*)-isomers leads to the conclusion that the (*E*,*Z*)-isomer content in the mixture is at least 30%.

The ^1^H NMR spectrum of 1,4-distyrylbenzene **1** (Figure 14b) recorded after irradiation is similar, but the intensity of the proton signals corresponding to the (*E*,*Z*)-isomer is much lower, and most of these signals are overlapped by those of the (*E*,*E*)-isomer. Two doublets for the α-H olefinic protons in the (*E*,*Z*)-isomer were unambiguously identified; they appear more upfield, at 6.37 ppm (*J* = 11.9 Hz) and 6.92 ppm (*J* = 16.2 Hz), while the intense doublet corresponding to the α-H proton in the (*E*,*E*)-isomer (*J* = 16.4 Hz) is at 6.94 ppm. Analysis of the integral intensities of proton signals corresponding to the (*E*,*E*)- and (*E*,*Z*)-isomers indicates that the content of the (*E*,Z)-isomer in the mixture does not exceed 10%.

Based on the ^1^H NMR data, we can conclude that isomerization involves one double bond and that the mixture contains both (*E*,*E*)- and (*E*,*Z*)-isomers. Distyrylbenzene **1** is less prone to *E*-*Z* photoisomerization, which is to be expected because the degree of conjugation between molecular groups is higher for the *para*-substituted derivative.

### 2.4. Quantum Chemical Calculations

The objectives of the quantum chemical calculation are (1) to calculate the energy differences of compounds **1** and **2** and their conformers in order to estimate the conformational composition, (2) to calculate λ_max_ in the absorption and fluorescence spectra, and (3) to elucidate, at the orbital levels the differences in the chromophore properties of these compounds. Table 3 illustrates the structures of the possible isomers and conformers (rotamers) of compounds **1** and **2** calculated with full opgeometry optimization at the B3LYP/DFT/D3BJ level of theory. The possible isomers include *trans*-*cis* (*E*–*Z*)-isomers relative to the double C=C bonds. The terms “*syn*” and “*anti*” stand for the conformers relative to the σ-bonds between the substituents and the central benzene ring. For the *Z*-isomers, only the mono-*Z*-isomers were calculated, since only one double bond was experimentally found to be isomerized upon irradiation of the initial trans forms **1** and **2**. The structures shown in Table 3 are two-dimensional projections constructed using calculated Cartesian atomic coordinates and provide a visual representation of the changing shape of the molecules.

Table 4 summarizes the energy and spectral parameters of the most favorable conformers and isomers.

It can be seen from Table 4 that the *trans*-forms of **1** and **2** in the ground S_0_ and excited S_1_ states are energetically the most favorable, with the energy differences between the *trans*-**1** and *trans*-**2** rotamers being low (0.13 and 0.04 kcal/mol, respectively) in comparison with *kT*. The relative contents of rotamers were calculated by the formula, as follows [22]:

(1 + exp(E_1_ − E_2_)/RT)^−1^ = n_1_/N (5)

where Δ*E* is the energy difference between the conformers, *k* is the Boltzmann constant, and *T* is the temperature (293 °C). This indicates that the presence of both conformers of *trans*-**1** in the equilibrium mixture amounts to 55% (in favor of *anti*,*anti*-(*E*,*E*)-**1**) and 45% for the other conformer, meaning that they are present in approximately equal amounts. As regards compound **2**, the energy difference between the conformers is 3.46 kcal/mol (Table 4). This is sufficient for *anti*,*anti*-(*E*,*E*)-**2** to predominate in the reaction mixture (99.7%). In the ^1^H NMR spectra discussed above, we observed the averaged proton signals for all possible conformers present in the solution.

**Figure 15:**
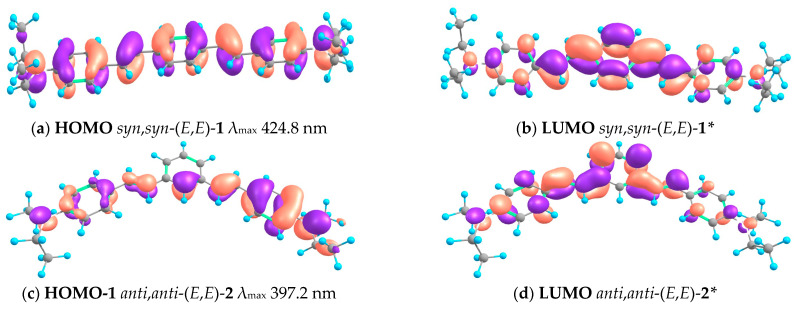
Structures of the frontier orbitals of *syn*,*syn*-(*E*,*E*)-**1** and *anti*,*anti*-(*E*,*E*)-**2** calculated at the B3LYP/DFT/D3BJ level. * indicates an excited state.

The results of the quantum chemical calculations of spectral parameters are in good agreement with the spectroscopic data. However, since TDDFT calculations do not result in the exact agreement between the calculated and experimental absorption and fluorescence λ_max_ values [23], we will discuss the trends in these values when moving from **1** to **2** and from *trans*- to *cis*-configuration. The calculation reproduced the blue shift of λ_max_ in the absorption spectra observed when moving from **1** to **2**, although the absolute values of calculated λ_max_ are somewhat overestimated. The blue shift of λ_max_ is due to the different orbital structures of **1** and **2**, caused by the different types of conjugation in *para*- and *meta*-isomers. The structures of the frontier MOs are shown in Figure 15.

Figure 15 demonstrates how the difference between the *para*- and *meta*-substitution is manifested. In the case of *syn*,*syn*-(*E*,*E*)-**1**, excitation involves an electron transition from the highest occupied MO (HOMO), which delocalizes over the whole molecule, to the lowest unoccupied MO (LUMO), the electron density maximum of which is concentrated mainly in the central part of the molecule and, to a lesser extent, on the dimethylaniline moieties. A different situation is observed for *anti*,*anti*-(*E*,*E*)-**2**. In this case, excitation induces migration of an electron from HOMO-1, the electron density maximum of which is concentrated in the right-hand part of the molecule and does not affect five C atoms of the central benzene ring, to LUMO, the electron density maximum of which is also concentrated in the central and left-hand parts. However, the main difference between the orbitals of **1** and **2** is that in the latter compound, there are nodal planes of HOMO-1 that pass through 1–6 and 3–4 C atoms of the central benzene ring and a LUMO nodal plane passing through the 2–5 C atoms of **2**. This means that the right and left halves of the molecule are isolated from each other. It is worth noting that in reality, this isolation leads to a partial loss of conjugation, manifested in a blue shift of the absorption band and estimated as 1.1 kcal/mol.

The calculation shows that the transition from *trans*- to *cis*-forms of **1** and **2** is accompanied by a blue shift of absorption bands (nm): from 425 to 408 and from 397 to 382, respectively.

## 3. Materials and Methods

Diethylamino 1,4- and 1,3-distyrylbenzenes (**1**, **2**) have been previously reported in the literature [24,25], but here they were synthesized by original procedures that will be published elsewhere. The structure and the purity of compounds **1** and **2** were confirmed by ^1^H NMR spectra. The structures with atom numbering differing from the IUPAC rules are shown in Figure 1. ^1^H NMR spectrum of **1** (Figure S1) and **2** (Figure S2) in MeCN are available in the Supplementary Material of this article.

The ^1^H NMR spectra were recorded on a Bruker (Billerica, MA, USA) DRX-500 spectrometer (500.13 MHz) in MeCN-*d*_3_ at 25 °C using residual solvent protons as the internal standard (δ_H_ 1.96 ppm). The chemical shifts were measured with an accuracy of 0.01 ppm, while the accuracy of measurement of the spin–spin coupling constant was 0.1 Hz.

Melting points were measured in capillaries on a Mel-Temp II device.

(*E*,*E*)-1,4-Bis(diethylaminodistyryl)benzene (**1**). *T*_m_ = 242–244 °C (cf. lit.: 242–246 °C) [16]. ^1^H NMR (500 MHz, MeCN-*d*_3_), 25 °C, δ: 1.16 (t, 12H, *J* = 7.0 Hz, 4*Me*CH_2_), 3.41 (q, 8H, *J* = 7.0 Hz, 4*CH_2_*Me), 6.72 (d, 4H, *J* = 8.8 Hz, 2 3′-H, 2 5′-H), 6.94 (d, 2H, *J* = 16.4 Hz, 2 α-H), 7.11 (d, 2H, *J* = 16.4 Hz, 2 β-H), 7.41 (d, 4H, *J* = 8.8 Hz,2 2′-H, 2 6′-H), 7.48 (br s, 4H, 2-H, 3-H, 5-H, 6-H).

(*E*,*E*)-1,3-Bis(diethylaminodistyryl)benzene (**2**). *T*_m_ = 136 °C. (cf. lit.: 134 °C) [17].

^1^H NMR (500 MHz, MeCN-*d*_3_) 25 °C, δ: 1.17 (t, 12H, *J* = 7.0 Hz, 4*Me*CH_2_), 3.42 (q, 8H, *J* = 7.0 Hz, 4*CH_2_*Me), 6.73 (d, 4H, *J* = 8.9 Hz, 2 3′-H, 2 5′-H), 6.96 (d, 2H, *J* = 16.3 Hz, 2 α-H), 7.16 (d, 2H, *J* = 16.3 Hz, 2 β-H), 7.30–7.36 (m, 3H, 4-H, 5-H, 6-H), 7.42 (d, 2H, *J* = 8.9 Hz, 2 2′-H, 2 6′-H), 7.66 (s, 1H, 2-H).

The absorption spectra were recorded on an Agilent (Santa Clara, CA, USA) 8453 spectrophotometer. The luminescence spectra were measured on a Varian Eclipse spectrofluorometer (Agilent, USA). Delayed fluorescence and phosphoresce were recorded at 200 µs after the light pulse used for excitation which ruled out the contribution of the fast fluorescence to the total recorded emission signal. The difference absorption spectra of photoreaction products and their transformation kinetics were measured on a nanosecond laser photolysis unit [23]. The irradiation was carried out with the 3rd harmonic of Nd-YAG laser (Solar) (*λ* = 355 nm). Dissolved oxygen was removed from the solution by bubbling argon through it. The fluorescence quantum yields were determined using the 9,10-diphenylanthracene as the standard (0.95 quantum yield in ethanol) [26]. The measurements were performed in MeCN (special purity grade, brand 0, Cryochrom, Saint Petersburg, Russia, Russia) at room temperature.

The ^1^H NMR experiment with irradiation of samples of compounds **1** and **2** was performed in the following way. First, the spectrum was recorded for a distyrylbenzene solution in MeCN-*d*_3_ prepared under red light, to prevent *trans-cis* isomerization. Then this solution was irradiated with unfiltered light of an F-SP-20-827-E27 fluorescent lamp for 30 min. After irradiation, the spectrum was recorded again.

Quantum chemical calculations for the isomers and conformers (rotamers) of molecules **1** and **2** in the ground and excited states were performed with full geometry optimization using B3LYP functional [27] at the DFT/def2-TZVP and TDDFT/def2-TZVP level of theory within the ORCA 5.0.3 software package [28]. The D3BJ dispersion correction [29] was also taken into account. The results of the calculations are summarized in Table 4.

## 4. Conclusions

The presence of two ethylene bonds in distyrylbenzenes **1** and **2** determines the existence of compounds as (*E*,*E*)- and (*E*,*Z*)-isomers. Their structures were confirmed by ^1^H NMR spectroscopy and studied using steady-state and laser kinetic spectroscopy methods. The transition from (*E*,*E*)-**1** to (*E*,*E*)-**2** is accompanied by a blue shift in the absorption and fluorescence maxima, attributable to different characters of π-conjugation in the molecules of **1** and **2**, as established by the analysis of their orbital structure. The blue shifts in the absorption and fluorescence maxima also take place upon transition from (*E*,*E*)- to (*E*,*Z*)-isomers (the latter were obtained through a photochemical route as stable species). For both compounds, photoexcitation is accompanied by *trans-cis* photoisomerization, intersystem crossing to the triplet state, and conventional (fast) and delayed fluorescence. The triplet–triplet (T–T) dismutation results in the formation of radical anions and radical cations with different lifetimes but similar absorption spectra. In the presence of an electron donor (ascorbic acid) and an electron acceptor (*para*-nitroacetophenone), electron transfer reactions take place, competing with the dismutation of triplet molecules. This gives rise to radical cations and radical anions, respectively. The highlighted photoprocesses of diethylamino distyrylbenzene derivatives can be used to design photoactive supramolecular systems involving macrocyclic compounds.

## Figures and Tables

**Table 1:** Absorption maxima for compounds **1** and **2**.

Compound	Experiment	Calculation
	λ_max_, nm	λ_max_, nm
**1**	408	425
**2**	373	397

**Table 2:** Fluorescence maxima, Stokes shifts, and fluorescence quantum yields of compounds **1** and **2**.

Compound	λ_max_, nm	λ_excit_, nm	ϕ	λ_0-0_, nm	Δλ, nm
**1**	500	410	0.56	452	90
**2**	470	370	0.24	414	100

**Table 3:** Structures of isomers and conformers of **1** and **2**.

Compound	Structure
*syn*,*syn*-(*E*,*E*)-**1**	* 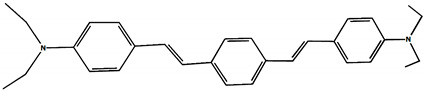 *
*syn*,*syn*-(*E*,*Z*)-**1**	* 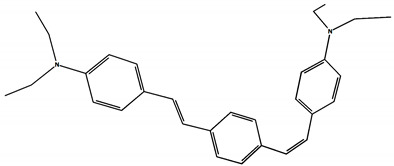 *
*anti*,*anti*-(*E*,*E*)-**1**	* 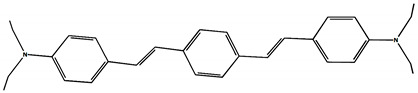 *
*anti*,*anti*-(*E*,*Z*)-**1**	* 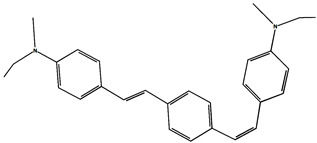 *
*anti*,*anti*-(*E*,*E*)-**2**	* 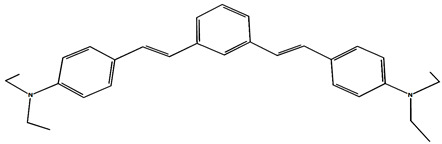 *
*anti*,*anti*-(*Z*,*E*)-**2**	* 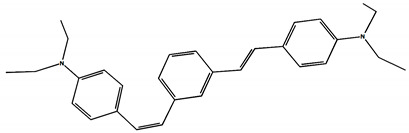 *
*syn*,*anti*-(*E*,*E*)-**2**	* 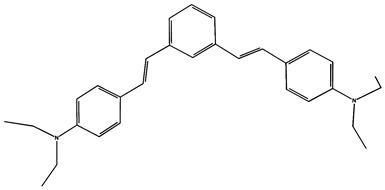 *
*syn*,*anti*-(*E*,*Z*)-**2**	* 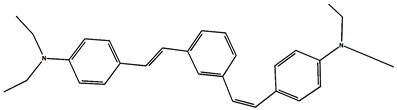 *
*syn*,*syn*-(*E*,*E*)-**2**	* 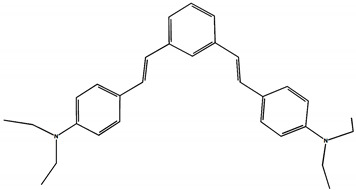 *
*syn*,*syn*-(*Z*,*E*)-**2**	* 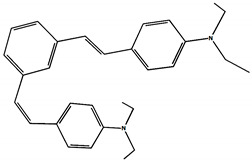 *

**Table 4:** Total energies of compounds **1** and **2** calculated at the B3LYP/DFT/D3BJ level, *E*_total_, energy differences between the isomers and conformers in the ground and excited states (*), Δ*E*, calculated absorption and fluorescence λ_max_.

Compounds	*E*_total_, a.u.	Δ*E*, kcal/mol	λ_max_, nm, abs.	λ_max_, nm flu.
*syn*,*syn*-(*E*,*E*)-**1**	−1274.12860	0.0 *^a^*	424.8	
*anti*,*anti*-(*E*,*E*)-**1**	−1274.12839	0.13	429.6	
*syn*,*syn*-(*E*,*Z*)-**1**	−1274.12256	3.95	418.3	
*syn*,*anti*-(*E*,*E*)-**2**	−1274.12715	0.0 *^a^*/1.07 *^b^*	387.6	
*anti*,*anti*-(*E*,*E*)-**2**	−1274.12709	0.04	397.2	
*syn*,*anti*-(*E*,*Z*)-**2**	−1274.12163	3.46	382.3	
*anti*,*anti*-(*E*,*E*)-1*	−1274.02644	0.0 *^a^*		462.9
*syn*,*syn*-(*E*,*E*)-1*	−1274.02535	0.68		457.0
*syn*,*syn*-(*E*,*Z*)-1*	−1274.02149	3.11		484.8
*anti*,*anti*-(*E*,*E*)-2*	−1274.01744	0.0 *^a^*/5.65 *^c^*		446.7
*syn*,*anti*-(*E*,*Z*)-2*	−1274.01111	3.97		457.6

*^a^* There are four groups of compounds, among which the energy of the most thermodynamically favorable ones is taken to be zero. *^b^* Energy difference between *syn*,*syn*-(*E*,*E*)-**1** and *syn*,*anti*-(*E*,*E*)-**2**. *^c^* Energy difference between *anti*,*anti*-(*E*,*E*)-1* and *anti*,*anti*-(*E*,*E*)-2*.

## Data Availability

Data are contained within the article and Supplementary Materials.

## Supplementary Materials

The following supporting information can be downloaded at: https://www.mdpi.com/article/10.3390/molecules29174139/s1, ^1^H NMR spectrum of (*E*,*E*)-1,4-bis(diethylaminodistyryl)benzene (**1**) (Figure S1) and (*E*,*E*)-1,3-bis(diethylaminodistyryl)benzene (**2**) (Figure S2) in MeCN are available in the Supplementary Material of this article.
