# Supplementary material for: Photoprocesses in Bis-Diethylamino Derivatives of 1,4- and 1,3-Distyrylbenzene
Source: Molecules. 2024 Aug 31;29(17):4139. doi: 10.3390/molecules29174139 (PMC11397533; doi:10.3390/molecules29174139)
Supplement: Supplementary file 1 [file molecules-29-04139-s001.zip › molecules-3172375-supplementary.pdf]

Supplementary Materials

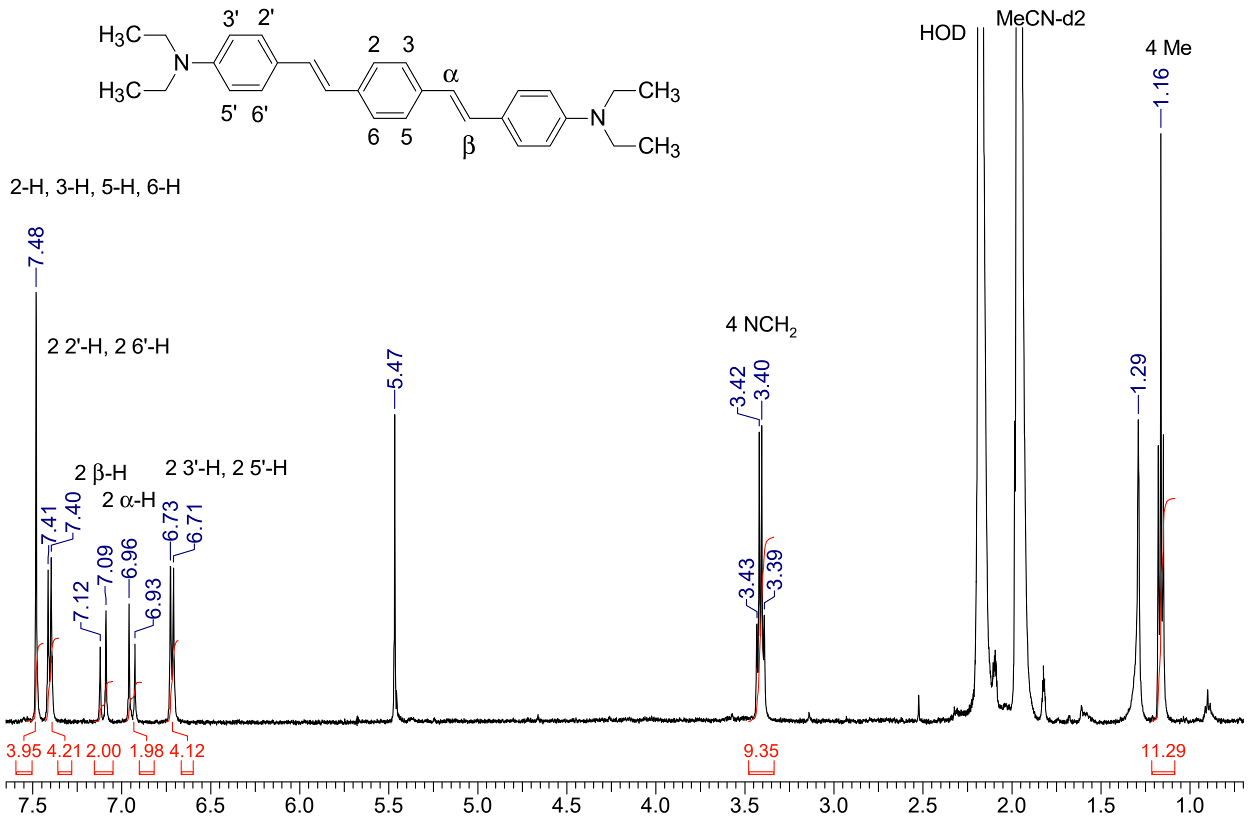

Figures S1. ^{1}H NMR spectrum of (*E*,*E*)-1,4-bis(diethylaminodistyryl)benzene (***1***) in MeCN-*d*$_3$, 25 °C.

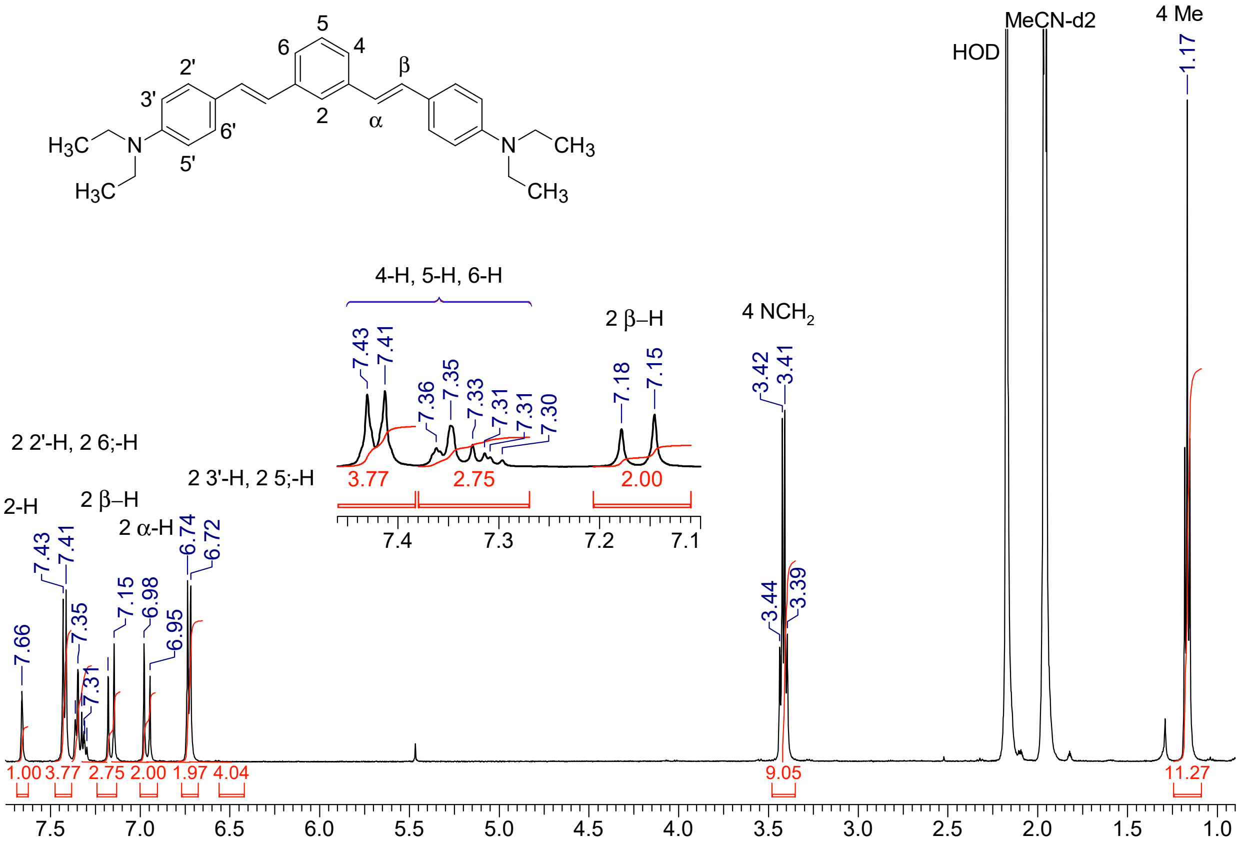

Figures S2. ^{1}H NMR spectrum of (*E*,*E*)-1,3-bis(diethylaminodistyryl)benzene (***2***) in MeCN-*d*$_3$, 25 °C.
